# Spontaneous recession of a posterior cerebral artery aneurysm concurrent with carotid rete mirabile and moyamoya-pattern collateral vessels: a case report

**DOI:** 10.1186/s12883-019-1277-7

**Published:** 2019-04-02

**Authors:** Hao Chen, Kun Hou, Xin Wang, Kan Xu, Jinlu Yu

**Affiliations:** Department of Neurosurgery, The First Hospital of Jilin University, No. 71, Xinmin Avenue, Changchun, 130021 China

**Keywords:** Carotid rete mirabile, Posterior cerebral artery, Moyamoya-pattern collateral vessel, Aneurysm, Conservative treatment

## Abstract

**Background:**

Carotid rete mirabile (RM) is a meshwork of multiple, freely intercommunicating arterioles that reconstitute the absent or hypoplastic segments of the internal carotid artery (ICA). Carotid RM has been reported to be associated with cerebrovascular diseases. However, it is rarely associated with moyamoya-pattern collateral vessels in the posterior cerebral artery (PCA) region and aneurysm.

**Case presentation:**

A 39-year-old woman was admitted complaining of sudden-onset headache, nausea, and vomiting. Further investigation revealed subarachnoid hemorrhage (SAH), carotid RM, a moyamoya collateral pattern in the PCA region, and a pseudoaneurysm in the moyamoya-like vessels. The patient was treated conservatively, recovered well and was discharged 1 week later. Follow-up angiography showed that the aneurysm had disappeared.

**Conclusions:**

As shown by the present case, we believe that carotid RM could occur in combination with moyamoya-pattern collateral vessels in the PCA region; aneurysms can occur in the moyamoya-like vascular network. Congenital etiology may be the reason for these combinations. Based on our approach in this case, aneurysm located in moyamoya-like vessels can disappear spontaneously after conservative treatment.

## Background

Carotid rete mirabile (RM), which is often found in lower mammals such as pigs and sheep and rarely occurs in humans, is a meshwork of multiple, freely intercommunicating arterioles that reconstitute the absent or hypoplastic segments of the internal carotid artery (ICA) [1]. Carotid RM has been reported to be associated with cerebrovascular diseases [1, 2]. However, it is rarely associated with a moyamoya-like collateral pattern of cerebral arteries.

Moyamoya-pattern collateral vessels mainly occur in the middle cerebral artery (MCA) region, presenting with characteristic hypertrophy and proliferation of the small arteries; these vessels serve as collateral channels to bypass occlusions [3, 4]. Rarely, the posterior cerebral artery (PCA) is also involved in moyamoya-pattern collateral vessels [5]. Aneurysms can occur in moyamoya-pattern collateral vessels [6]. The natural history of aneurysms in moyamoya-pattern collateral vessels is uncertain.

Here, we report a rare case of a patient who presented with carotid RM, moyamoya-pattern collateral vessels in the PCA region and an aneurysm that was located in the collateral network. The aneurysm regressed after conservative treatment. The combination of carotid RM, moyamoya-pattern collateral vessels in the PCA region and aneurysm is discussed in the paper.

## Case presentation

A 39-year-old woman was admitted complaining of sudden-onset headache, nausea, and vomiting for 1 day. She had a history of hypertension for 10 years and denied any history of diabetes, hyperlipidemia, autoimmune diseases, or other remarkable illnesses. She was alert and could correctly obey commands. Physical examination was unremarkable except for neck stiffness. Her blood glucose, electrolytes, blood cell counts, coagulation test, and antinuclear antibody series were within normal limits during laboratory investigations.

**Fig. 1:**
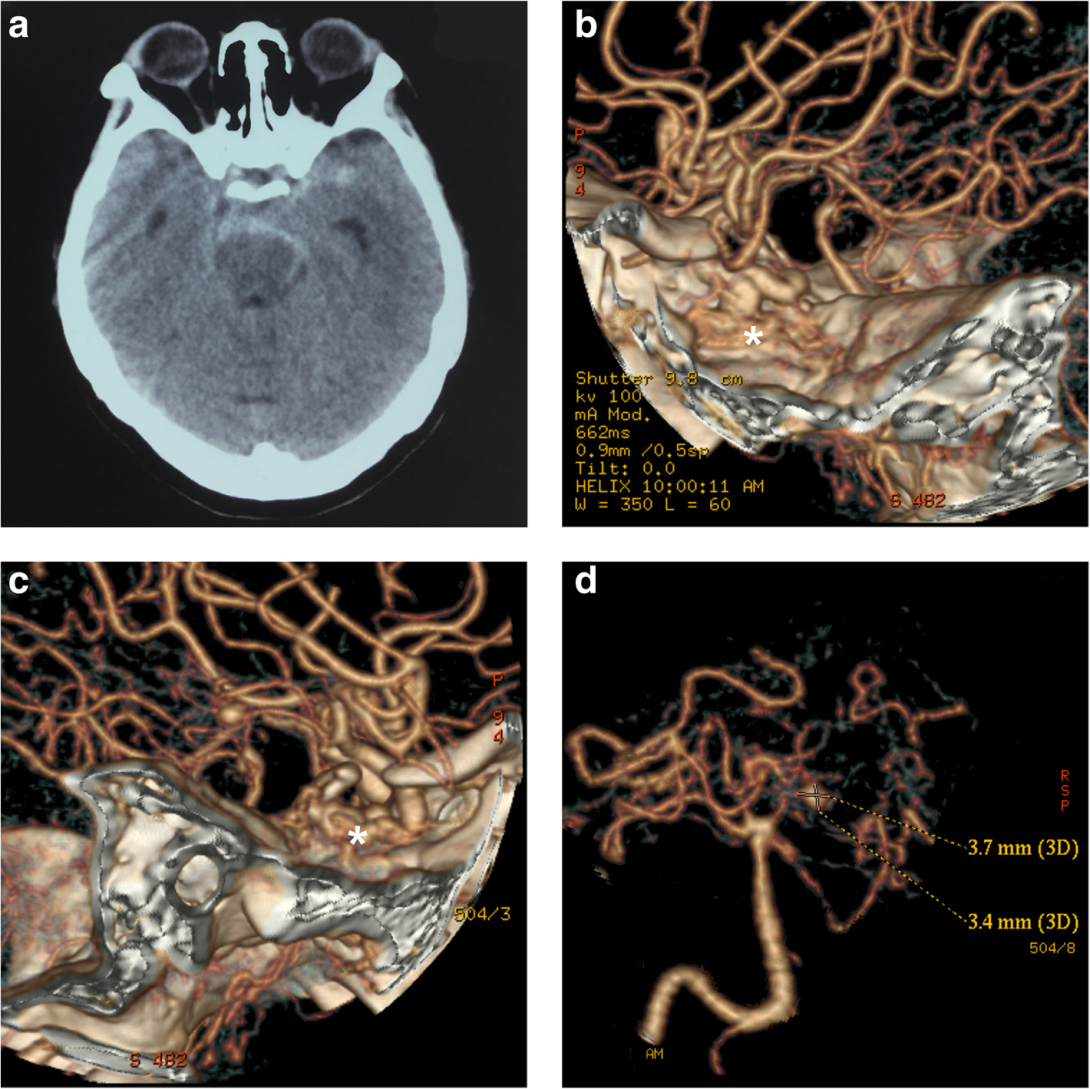
**a**: A head CT scan shows an SAH concentrated in the perimesencephalic cistern. **b**-**c**: CTA shows that the cavernous portion of the bilateral internal carotid arteries (ICAs) is absent (asterisks). The bilateral middle cerebral arteries and anterior arteries are normal. **d**: An aneurysm is identified in the PCA region. Abbreviations: CT: computed tomography, SAH: subarachnoid hemorrhage, CTA: CT angiography, ICA: internal carotid artery, PCA: posterior cerebral artery

Head computed tomography (CT) showed a subarachnoid hemorrhage (SAH) concentrated in the perimesencephalic cistern (Fig. 1a). CT angiography (CTA) showed that the bilateral ICAs were absent in the skull base (Fig. 1b-c). The bilateral MCAs and anterior arteries were normal. An aneurysm was identified in the PCA region (Fig. 1d).

**Fig. 2:**
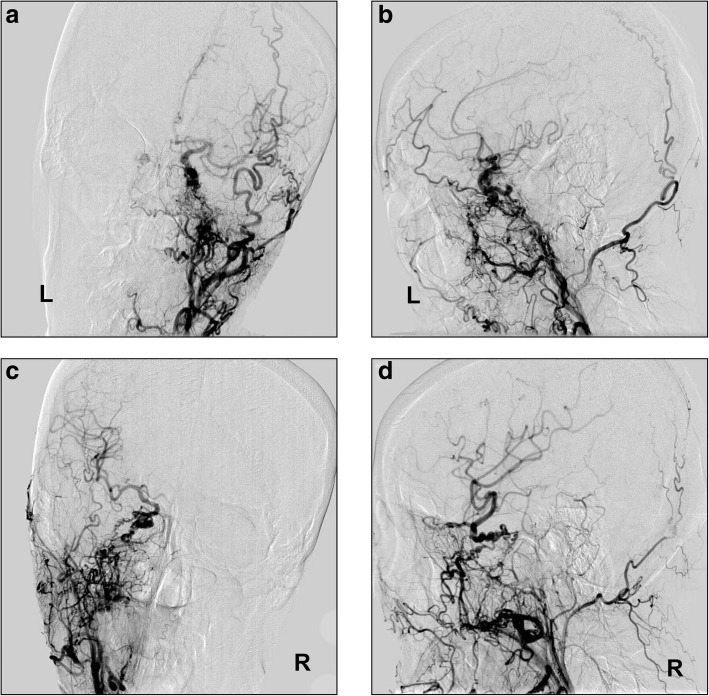
**a**-**b**: Left common carotid artery DSA in AP and lateral views shows that the internal maxillary artery and ascending pharyngeal artery of the external carotid artery are confluent with the cavernous portion of the ICA at the skull base. And the ICA above the cavernous portion segment, middle cerebral artery, and anterior cerebral artery are normal in shape and direction. **c**-**d**: The findings of right common carotid artery DSA are similar to those of the left common carotid artery. Abbreviations: AP: anteroposterior, DSA: digital subtraction angiography, ICA: internal carotid artery

**Fig. 3:**
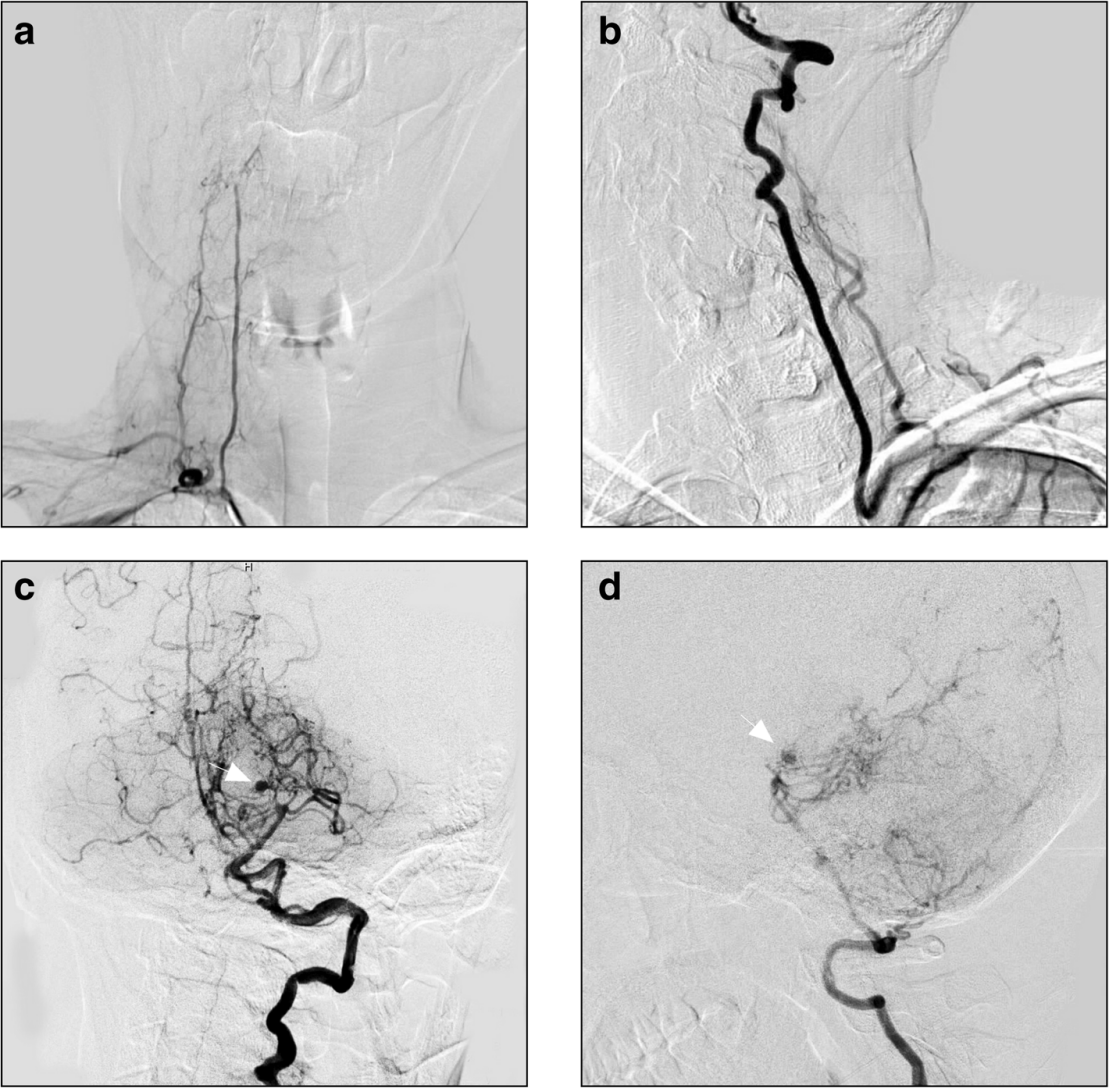
**a**-**b**: Right subclavian artery angiogram shows the right VA is slender. Left subclavian artery angiogram shows the left VA is well developed. **c**-**d**: Left VA angiogram in AP and lateral views shows that the left VA is extended tortuously to the basilar artery; the distal vessels of the bilateral PCAs are fine or even absent, and the capillaries around this area have compensatory hyperplasia. An aneurysm is clearly visible in the left moyamoya-pattern collateral vessels (arrows). Abbreviations: AP: anteroposterior, PCA: posterior cerebral artery, VA: vertebral artery

Head digital subtraction angiography (DSA) showed that the bilateral internal maxillary arteries and ascending pharyngeal arteries were confluent with the cavernous portion of the ICAs at the skull base. And the bilateral ICAs above the cavernous portion of the ICAs, the MCAs, and the anterior cerebral arteries were normal (Fig. 2). The right vertebral artery (VA) was fine, while the left VA was well developed (Fig. 3a-b). Moyamoya-pattern collateral vessels could be seen in the bilateral PCAs region, the distal part of the PCA was composed of abnormally fine vessels, and an aneurysm was clearly identified in the left moyamoya-pattern collateral vessels (Fig. 3c-d).

**Fig. 4:**
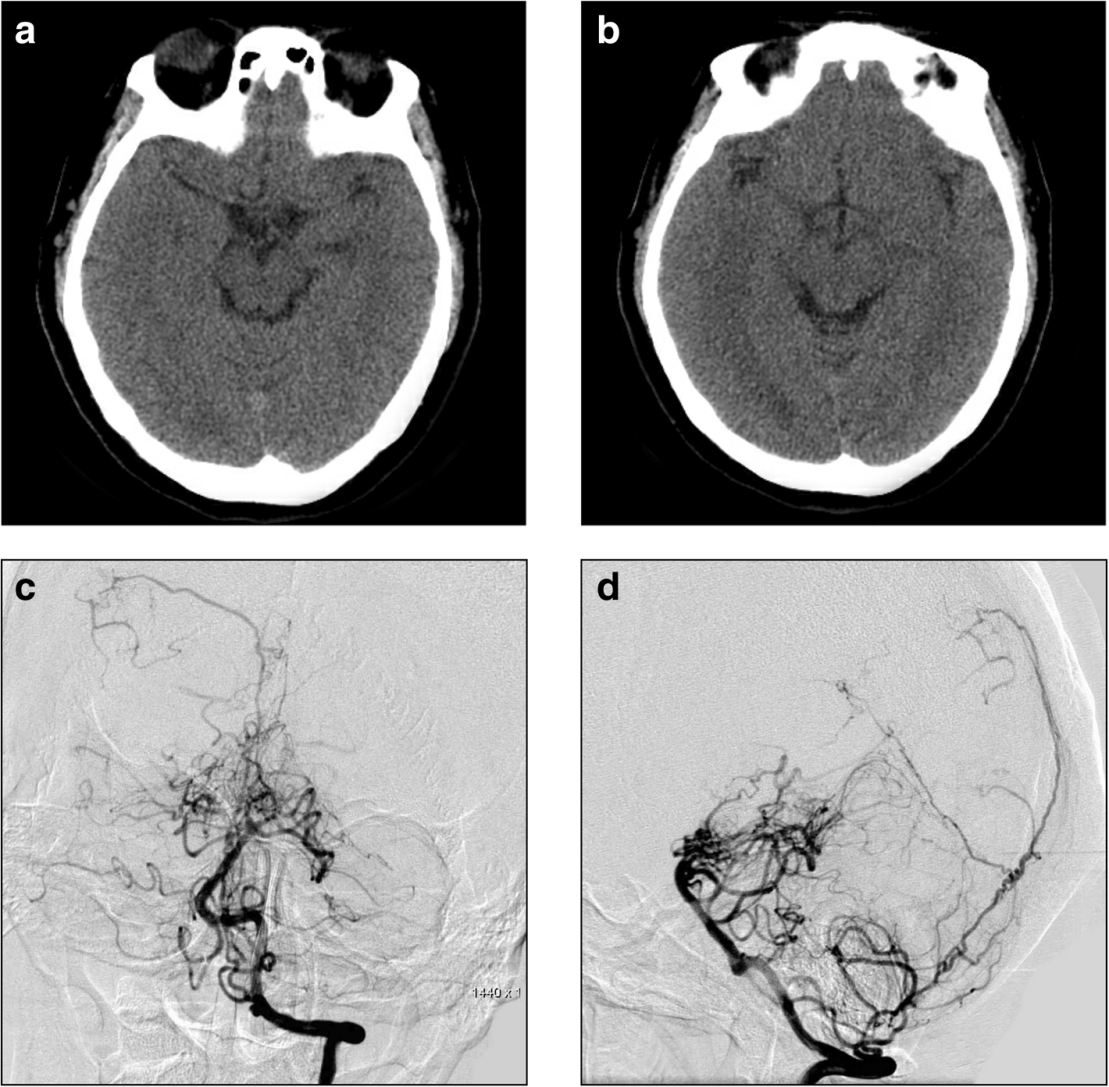
**a**-**b**: CT shows complete resolution of the SAH. **c**-**d**: Left VA angiogram in AP and lateral views reveals that the aneurysm have disappeared, the distal part of the PCAs become finer and smaller, and the transdural compensation of the posterior meningeal artery could be seen. Abbreviations: AP: anteroposterior; CT: computed tomography, SAH: subarachnoid hemorrhage, PCA: posterior cerebral artery, VA: vertebral artery

She was diagnosed as bilateral carotid RM, moyamoya-like vessels in the PCA region, intracranial pseudoaneurysm, and SAH. Management of the aneurysm was difficult, and wait and see management was adopted. The patient experienced an uneventful recovery and was discharged 1 week later. Follow-up CT performed 44 days later showed complete resolution of the SAH (Fig. 4a-b). Head DSA revealed that the pseudoaneurysm disappeared spontaneously while the moyamoya-pattern collateral vessels were unchanged, the distal part of the PCAs became finer and smaller, and the transdural compensation of the posterior meningeal artery was visible (Fig. 4c-d). The patient was in good condition during follow-up, she returned to her normal activities.

## Discussion and conclusion

Carotid RM refers to a meshwork of multiple, freely intercommunicating fine arteries or arterioles that reconstitute the absent or hypoplastic segments of the ICA and is often found in lower mammals [1]. In rare cases, humans can also develop carotid RM. The diagnostic criteria for carotid RM include the following: 1) a hypoplastic ICA, 2) an arterial plexus between the maxillary artery and the cavernous portion of the ICA; 3) a dilated ophthalmic artery (OA); 4) a supraclinoid ICA that is not hypoplastic and is fed by the arterial plexus and the OA; 5) bilateral lesions; and 6) no abnormal vessels, such as moyamoya-like vessels, in the intradural circulation [7, 8]. The case reported in this paper met all of the carotid RM diagnostic criteria.

Carotid RM has been reported to be associated with many cerebrovascular diseases, such as intracranial aneurysm, arteriovenous malformation, pial arteriovenous fistula, carotid-cavernous fistula, and Galen aneurysmal malformation [1, 2, 9, 10]. However, carotid RM is rarely associated with a moyamoya-pattern collateral vessels of the posterior circulation. Moyamoya-pattern collateral vessels mainly occur in the MCA region [3, 4]. Rarely, the PCA could be also involved in moyamoya-pattern collateral vessels, as in our case [5].

The reasons that concomitant cerebrovascular diseases are associated with carotid RM are unclear. Currently, these combinations are thought to arise from congenital embryonic etiologies [11]. Therefore, congenital etiology may also be the reason that carotid RM was associated with a moyamoya-pattern collateral vessels of the PCA in our case.

Most patients with isolated moyamoya-pattern collateral vessels do not have severe perfusion impairment [3]. In our case, the moyamoya-pattern collateral vessels were visible in the bilateral PCAs region, and the transdural compensation from the posterior meningeal artery to the distal part of the PCA was also visible. Due to the sufficient collateral circulation, the CT scan did not show signs of ischemia in the territory of vertebrobasilar artery system (Figs. 1 and 4). The posterior meningeal artery transdural anastomosis observed in our case is rare, and the compensation pattern was the same as that seen in the middle meningeal artery in moyamoya disease [12, 13].

When moyamoya-pattern collateral vessels occur in the PCA region, the rich moyamoya-like vascular network may be associated with an unfavorable outcome because these expanded moyamoya-like vessels endure higher hemodynamic stress, which can easily result in aneurysm formation [14, 15]. These aneurysms can easily rupture. The complex anatomy increases the difficulty of treating aneurysms located in the moyamoya-like vascular network. Although successful treatment has been reported, the risk is very high [6, 16].

It is uncertain whether aneurysms in moyamoya-pattern collateral vessels should be treated due to a lack of understanding of their natural history. Kawaguchi et al. reported that these aneurysms of the moyamoya-like vascular network can disappear spontaneously after the initial bleeding episode [15]. Our previous study also found that pseudoaneurysms can disappear after conservative treatment [17]. Because of the uncertainty regarding the natural history of aneurysms in moyamoya-like vessels, we chose conservative treatment for our patient. The aneurysm had disappeared on follow-up DSA performed at 44 days later.

As shown by the present case, we believe that carotid RM can occur combined with moyamoya-pattern collateral vessels in the PCA region. And aneurysms can occur in the moyamoya-like vascular network. Furthermore, based on our approach in this case, aneurysms located in moyamoya-like vessels can also disappear spontaneously after conservative treatment.

## Abbreviations

CT: Computed tomography
CTA: CT angiography
DSA: Digital subtraction angiography
ICA: Internal carotid artery
MCA: Middle cerebral artery
OA: Ophthalmic artery
PCA: Posterior cerebral artery
RM: Rete mirabile
SAH: Subarachnoid hemorrhage
VA: Vertebral artery
